# Ion channel mutations and cancer

**DOI:** 10.1016/j.bbrep.2025.101990

**Published:** 2025-04-03

**Authors:** Xinyu Cao, Liang Yan, Liang Hong

**Affiliations:** a Department of Medicine, University of Illinois at Chicago, Chicago, IL, 60612, USA; b Department of Biomedical Engineering, University of Illinois at Chicago, Chicago, IL, 60612, USA; c Department of Physiology and Biophysics, University of Illinois at Chicago, Chicago, IL, 60612, USA

**Keywords:** Ion channel, Mutation, Variant, Gene, Cancer

## Abstract

Cancer is characterized by uncontrolled growth and spread of abnormal cells, driven by genetic, environmental, and lifestyle factors. Genetic mutations contribute to hallmark processes of cancer progression such as sustained proliferation, apoptosis resistance, and immune evasion. Ion channels are pore-forming transmembrane proteins that regulate ion transport across cellular membranes, influencing various cellular functions. Recent studies have indicated the emerging roles of ion channel proteins in cancer. Ion channels are critical for cellular processes like proliferation, apoptosis, migration, and angiogenesis, and dysregulation of ion channels by genetic mutations disrupts these processes, enabling tumor growth, invasion, and metastasis. Ion channel gene mutations have been associated with various cancer subtypes. These ion channel mutations either dysregulate ion channel activity associated with intracellular signaling pathways in cell survival and division, or influence the tumor microenvironment by modifying pH, oxygenation, or ion concentrations, which might facilitate tumor growth and gene expression and contribute to oncogenesis. In the present review, we discuss ion channel regulation of cancer biology and summarize recent studies in ion channel mutations associated with cancer.

## Highlights

• Mutations in the genes are one of the major factors contributing to cancer progression.
• Ion channel function is critical for cellular processes.
• Ion channel mutations have been associated with various cancer subtypes.
• Ion channel mutations are novel therapeutic targets in cancers.

## 1 Introduction

Cancer is a severe disease characterized by the uncontrolled growth and spread of abnormal cells. It is one of the leading causes of mortality worldwide. Cancer cells exhibit several distinct properties including uncontrolled proliferation, sustained angiogenesis, and invasion and metastasis. These properties, often referred to as the "hallmarks of cancer," allow cancer cells to grow uncontrollably, evade normal regulatory mechanisms, and spread throughout the body.

Cancer is caused by a combination of genetic, environmental, and lifestyle factors that lead to the transformation of normal cells into malignant ones. While genetic factors play a critical role, an increasing body of evidence highlights the impact of modifiable factors such as diet, physical activity, smoking, alcohol consumption, and exposure to environmental pollutants (e.g., carcinogens, radiation exposure, toxins, etc.) [1,2]. Mutations in key genes are one of the major genetic factors contributing to cancer progression. Genetic mutations disrupt normal cellular processes, contributing to key hallmarks of cancer, including sustained proliferative signaling, resistance to cell death, and immune evasion. Mutations in oncogenes, such as EGFR (Epidermal growth factor receptor), result in constitutive activation, driving uncontrolled cell growth and division [3,4]. Additionally, loss-of-function mutations in tumor suppressors like TP53 (Tumor protein P53) and PTEN (Phosphatase and tensin homologue) eliminate critical regulatory mechanisms that restrain cell proliferation and survival, thereby facilitating tumorigenesis [[5], [6], [7]]. In addition to genetic mutations in these proteins linked with cancer progression, recent studies have identified that mutations in ion channels were implicated in cancer. In the present review, we discuss the ion channel protein regulation of cancer biology and summarize studies in ion channel mutations associated with the pathogenesis of cancer.

## 2 Ion channel and cancer

**Fig. 1:**
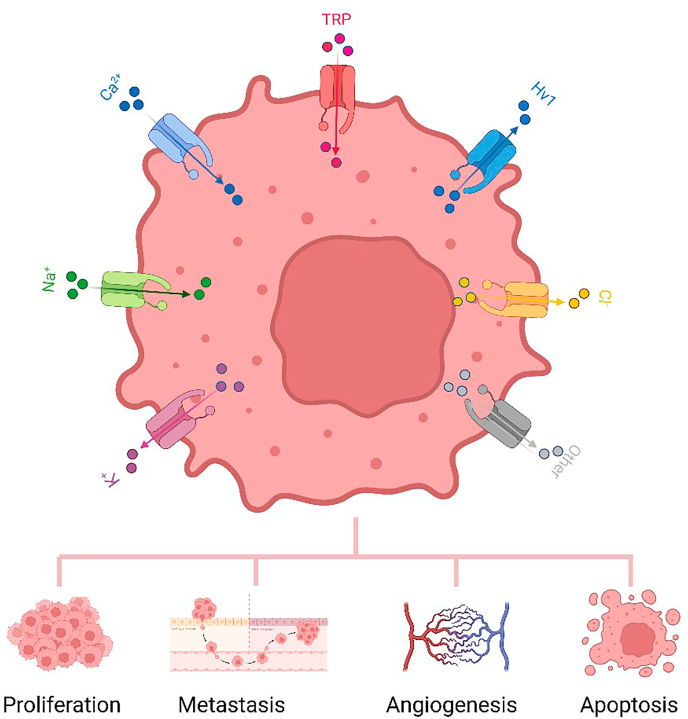
**Ion channels linked to cancer biology.** Ion channels, including potassium (K^+^), sodium (Na^+^), calcium (Ca^2+^), hydrogen (H^+^), chloride (Cl^−^), and transient receptor potential (TRP) channels, play critical roles in cancer progression. Dysregulation of these channels affects key cellular processes such as proliferation, metastasis, angiogenesis, apoptosis, and metabolic adaptation.

Ion channels are pore-forming transmembrane proteins that regulate ion transport across cellular membranes, influencing a wide range of cellular functions, including electrical excitation, movement, and metabolic processes [[8], [9], [10], [11], [12]]. The ion flow through each type of ion channel is essential for its physiological function, playing a critical role in maintaining tissue homeostasis, including cell proliferation, migration, and apoptosis (Fig. 1). When the expression or function of these channels becomes aberrant, it can disrupt these processes, leading to the transformation of normal cells into malignant ones that proliferate uncontrollably and spread, driving carcinogenesis [13].

**Fig. 2:**
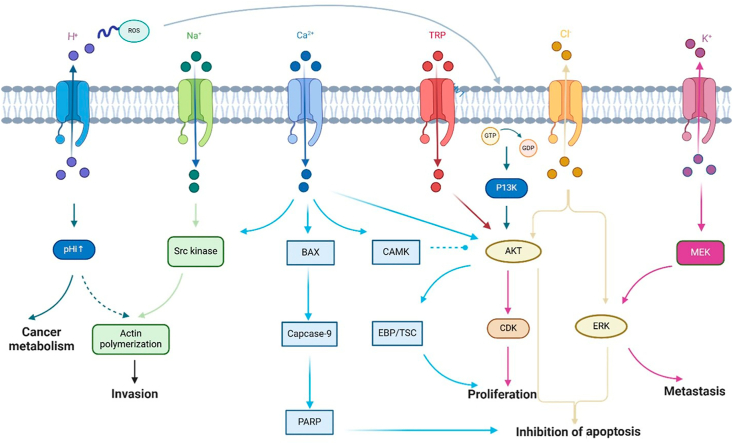
**Ion channel-mediated signaling pathways in carcer biology.** H^+^ channel can enhance the protons extrusion activity resulting intracellular alkalinization, which can help to maintain cancer cell metabolism. Na^+^ channel activity sustains Src kinase activity, which enhances actin nucleation and filament polymerization to modify cancer invasion. Ca^2+^ channel mediate Bcl-2-associated X protein (BAX) and calcium/calmodulin-dependent protein kinase (CAMK) signaling pathways to regulate apoptosis and proliferation in cancer development. TRP channels regulate AKT/ERK pathway. K^+^ channel promotes phosphorylation of MEK and thus activate ERK, which plays an important role in cancer cell's metastasis.

Ion channels influence cell proliferation, migration, invasion, apoptosis, and interactions with the tumor microenvironment (Fig. 2). These effects can be categorized into conductive and non-conductive functions, both of which contribute to tumor development and progression.

Conductive functions are the classical role of ion channels in regulating ion fluxes across the plasma membrane and organelle membranes, modulating cell homeostasis and signaling. For example, potassium (K^+^) channels, sodium (Na^+^) channels, and calcium (Ca^2+^) channels are critical for maintaining membrane potential, which influence cancer cell proliferation [[14], [15], [16], [17], [18], [19]]. Ion channels also modify cell apoptosis, aberrant K^+^ efflux, and Ca^2+^ and Na ^+^ influx have been linked to resistance to apoptotic signaling in cancer cells, promoting their survival under stress conditions [[20], [21], [22]]. Moreover, chloride (Cl^−^) channels and proton (H^+^) channels play essential roles in cell volume regulation and pH regulation, which facilitate cancer cell metabolism [[23], [24], [25]].

In addition to ion transport, ion channels carry out non-conductive functions by interacting with cytoskeletal elements and modulating intracellular signaling pathways. Ion channels interact with intracellular proteins and influence cell migration and invasion by modulating cytoskeletal dynamics and cell-matrix adhesion [[26], [27], [28]]. Ion channels also influence gene expression through non-conductive mechanisms. For instance, the voltage-gated Na^+^ channel is a key regulator of a gene transcriptional network controlling colon cancer invasion [29]. Moreover, endothelial ion channels regulate angiogenic signaling pathways that sustain tumor growth [30,31].

## 3 Ion channel mutations in cancer

**Fig. 3:**
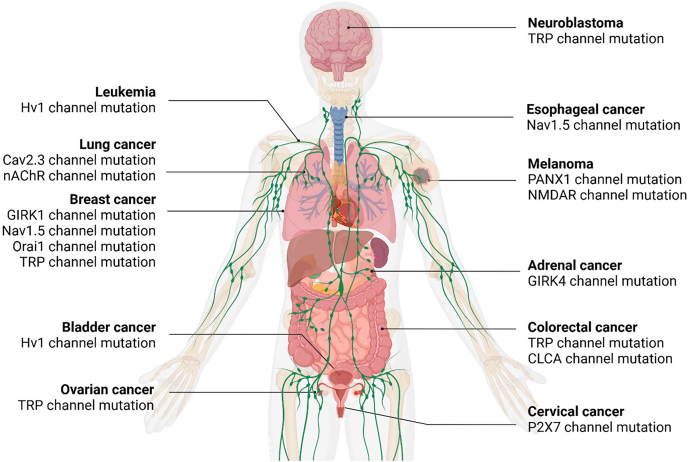
**Ion channel mutations in cancers.** The figure summarizes channel mutations associated with cancers that were reported in recent literature. GIRK K^+^ channel mutations are identified in breast and adrenal cancers. Nav1.5 Na ^+^ channel mutations are associated with breast and esophageal cancers. Cav2.3 Ca^2+^ channel mutations are linked with lung cancer. Orai1 Ca^2+^ channel mutations are identified in breast cancer. Hv1 H^+^ channel mutations are identified in leukemia and bladder cancer. TRP channel mutations are implicated in neuroblastoma, breast, colorectal, and ovarian cancers. P2X channel mutations are associated with cervical cancer. PANX1 channel mutations are identified in breast cancer and melanoma. *CLCA* mutations are implicated in colorectal cancer. nAChR mutations are associated with lung cancer. NMDAR mutations are identified in melanoma.

Ion channel gene mutations are associated with various cancer subtypes (Fig. 3). Potassium channel mutations were observed in breast cancer and adrenal cancer, sodium channel gene variants were identified in colorectal and gastric cancers, and calcium channel mutations were implicated in lung cancer. Moreover, mutations in TRP (Transient receptor potential channel) were found in neuroblastoma and ovarian cancer, and proton channel mutations were linked with leukemia and bladder cancer. These ion channel mutations might either dysregulate ion channel activity underlying cellular signaling pathways essential for cell survival and division, or influence the tumor microenvironment by modifying pH, oxygenation, or ion concentrations, which modify tumor growth and immune evasion. Additionally, ion channel mutations are associated with changes in gene expression and chromatin remodeling, contributing to oncogenesis. Although most ion channel mutations were identified in cancer patients, some were also found in cancer cell lines. Mutations in patients were typically identified through the analysis of primary or metastatic tumor tissues. These mutations arise through genetic and epigenetic alterations. In contrast, cancer cell lines were derived from patient tumors but underwent extensive adaptation to in vitro conditions, leading to the acquisition of additional mutations.

### 3.1 K^+^ channel mutations in cancer

The G protein-coupled inwardly rectifying potassium channels (GIRKs) are a family of inward-rectifier potassium (K^+^) ion channels. The GIRK1 channel (encoded by the *KCNJ3* gene) variants have been identified in both cancer cell lines and patients [[32], [33], [34]]. GIRK1 channel variants were reported to regulate cancer progression in breast cancer cell lines [32,33]. GIRK1 can interact with GIRK4 (encoded by the *KCNJ5* gene) to form functional heterooligomeric GIRK channels [33]. Functional analysis through heterologous expressions in *Xenopus laevis* oocytes showed that only the GIRK1a subunit could form functional GIRK channels in combination with hGIRK4. The other splice variants (GIRK1c, GIRK1d, and GIRK1e), while expressed, exhibited a dominant-negative effect on heterooligomeric channel function. These variants differ primarily in their cytosolic C-terminal region, which is critical for G-protein activation and phosphorylation, suggesting a pathophysiological role of these variants in breast cancer [32,33]. It is noted that overexpression of GIRK1a and GIRK1c in breast cancer cell line MCF7 cells increased cancer migration and proliferation [33]. Moreover, Pelzmann et al. characterized *KCNJ3* mutations associated with primary malign tumors [34]. They found that some mutations (e.g., E140 M, A142T, M184I) showed increased expression as well as increased function, and some (e.g., I151 N, G158S) presented minuscule expression but increased function relative to expression levels. The data indicated that these gain of function mutations in the *KCNJ3* gene may contribute to the development and progression of malignancies in tissues [34]. In addition to *KCNJ3*, mutations in *KCNJ5* (G151R and L168R) were shown to play essential roles in adrenal tumors [35].

### 3.2 Na ^+^ channel mutations in cancer

The voltage-gated sodium channel Nav1.5 (encoded by the *SCN5A* gene) was reported in breast cancer cell lines and patients with breast cancer [[36], [37], [38]]. Brackenbury et al. characterized a cancer-related neonatal splice variant of Nav1.5 [36]. The neonatal variant of Nav1.5 was shown to be primarily responsible for the voltage-gated sodium channel-dependent enhancement of invasive behaviors in MDAMB-231 breast cancer cells. And siRNA-mediated knockdown of the Nav1.5 neonatal variant reduced breast cancer cell migration by 43 % [36]. The data suggested that the neonatal variant Nav1.5 channel is a new target for metastatic breast cancer. Moreover, the cancer-related neonatal variant Nav1.5 channel exhibited a pharmacological distinctiveness compared to the wild-type Nav1.5 channel [38]. Fraser et al. characterized that one mutant in the neonatal variant Nav1.5 channel (K211D) plays a key role in the pharmacological distinctions [38]. Additionally, another Nav1.5 mutation H558R was identified in esophageal cancer patients [37].

### 3.3 Ca^2+^ channel mutations in cancer

There are several Ca^2+^ channel mutations linked with tumor development. It was reported that voltage-gated calcium channel Ca_v_2.3 mutations were implicated in lung cancer patients [39]. The Ca_v_2.3 is encoded by the *CACNA1E* gene. Gao et al. found that *CACNA1E* gene mutations A275S and R249G play significant roles in non-small cell lung cancer (NSCLC) [39]. Overexpression of A275S and R249G mutations enhanced NSCLC cell proliferation by activating the epidermal growth factor receptor (EGFR) signaling pathway [39]. Moreover, studies identified Orai1 calcium channel variants in MCF7 breast cancer cells [40], and it was shown that Orai1 variant Orai1α is essential for constitutive Ca^2+^ entry in MCF7 cancer cells, indicating a potential target for the formation of mammary microcalcifications in luminal breast cancer [40].

### 3.4 TRP channel mutations in cancer

TRPC and TRPM channel variants were reported to be linked with cancer development. TRPC splice variants in SKOV-3 ovarian cancer cells were reported to promote ovarian cancer cell proliferation and tumorigenesis [41]. Zeng et al. observed high expression levels of spliced variants TRPC1β, TRPC3a, TRPC4β, TRPC4γ, and TRPC6 (with exon 3 and 4 deletions), as well as a novel TRPC1 isoform with exon 9 deletion (TRPC1(E9_del_)) in human ovarian adenocarcinoma and ovarian adenocarcinoma-derived cell line SKOV3 [41]. Overexpression of TRPC variants in SKOV3 ovarian cancer cells significantly promoted cancer colony growth [41]. Moreover, Chen et al. identified that splice variants of TRPM2 channel are required for neuroblastoma tumor growth in patients [42]. In neuroblastoma, the TRPM2 variant TRPM2-L protected cells from oxidative stress by increasing levels of FOXO3a (a transcription factor) and superoxide dismutase 2, which help reduced the production of reactive oxygen species (ROS) and prevented oxidative cell death [43]. However, the shorter isoform, TRPM2-S, downregulated FOXO3a and superoxide dismutase 2 levels, impaired calcium influx in response to oxidative stress, and increased ROS, leading to reduced cell viability. In addition, tumors expressing TRPM2-S grew significantly slower when compared to those expressing TRPM2-L. TRPM2-S expression led to a reduction in hypoxia-inducible factors (HIF)-1/2α and downstream targets such as proteins involved in glycolysis, angiogenesis, mitochondrial function, and oxidative stress. Additionally, inhibiting TRPM2-L or expressing TRPM2-S increased sensitivity to doxorubicin. These findings suggest that TRPM2 plays a crucial role in tumor growth and survival, and targeting TRPM2-L could be a potential strategy to reduce tumor growth and enhance chemotherapy efficacy by modulating HIF-1/2 and mitochondrial function [42]. Another study reported that a splice variant in TRPM4 predisposed to familial colorectal cancer [44]. The splice site variant (TRPM4 c.25-1 G > T) leads to a frameshift and a premature protein truncation of 17 codons downstream (p. Ser9Leufs∗17), which might generate a defect of the TRPM4 channel. Moreover, mutations in TRPM7 (T1482I), TRPV4 (P19S), and TRPV6 (S18A, M721T, M418V, C197R) were identified in breast cancer and colorectal cancer patients [45].

### 3.5 H^+^ channel mutations in cancer

The voltage-gated proton channel Hv1 plays an essential role in cellular pH regulation. Several key elements in the Hv1 proton channel play crucial roles in the regulation of the channel function [[46], [47], [48]]. A key role of Hv1 channels is to support pH homeostasis, which is critical for proper cellular function [49]. The voltage-gated proton channel Hv1 was reported to be involved in the process of tumor progression [50], and H^+^ efflux mediated by the Hv1 channel in breast cancer cells was proposed to enhance intracellular alkalinization facilitating cancer cell metabolism [23,24]. Hv1 variants were linked with human diseases. A shorter isoform of Hv1 (HVCN1s), which lacks the first 20 amino residues, was enriched in malignant B lymphocytes from patients with chronic lymphocytic leukemia [51]. The B cells with a shorter isoform of Hv1 displayed enhanced proton currents and greater proliferation and migration. The properties of HVCN1s suggest that it may contribute to the pathogenesis of BCR-dependent B-cell malignancies [51]. Moreover, one Hv1 mutation F150C was identified in bladder carcinoma, and the mutation produced a perturbation of the channel function [52].

### 3.6 Other ion channel mutations in cancer

#### 3.6.1 P2X7 mutations

The P2X7 receptor is a ligand-gated ion channel that is activated in high concentrations of extracellular adenosine triphosphate (ATP). A truncated variant of the human P2X7 receptor was identified in human cervical cancer cell lines [53]. The variant, named P2X7-j, lacks the intracellular carboxyl terminus, the second transmembrane domain, and part of the extracellular loop. P2X7-j is expressed on the plasma membrane but shows diminished ligand-binding and channel function and is unable to form pores or mediate apoptosis in response to the P2X7 agonist benzoyl-ATP. P2X7-j interacts with the full-length P2X7 receptor and inhibits P2X7-mediated functions. These findings identified that P2X7-j is a novel variant associated with cervical cancer progression [53].

#### 3.6.2 PANX1 mutations

PANX1 is a glycoprotein involved in cellular communication through large pore channels that are permeable to ions and ATP. PANX1 inhibition reduces tumorigenic and metastatic properties. PANX1 genetic variants were identified in tumors of melanoma patients and cancer cell lines [54]. Nouri-Nejad et al. discovered that PANX1 mutations (Q5H and Y150F) contributed to breast cancer and melanoma. The mutations prevented phosphorylation at Y150 and altered N-glycosylation, potentially affecting melanoma progression [54].

#### 3.6.3 CLCA mutations

Chloride channel calcium-activated (*CLCA*) genes encode proteins that regulate chloride transport across cell membranes. Mo et al. identified frameshift mutations in *CLCA4* (c.218delT, c.218dupT, and c.544delA) in colorectal cancer patients [55], and showed that the mutations were associated with the tumorigenesis of the cancer [55].

#### 3.6.4 nAChR mutations

The acetylcholine receptor (AChR), known as a cholinergic receptor, is an integral membrane protein that responds to the neurotransmitter acetylcholine. A subtype of these receptors, nicotinic acetylcholine receptors (nAChRs), also called "ionotropic" acetylcholine receptors, are especially sensitive to nicotine. These receptors function as ion channels. A D398 N mutation in the α5 subunit of nAChRs was reported in lung cancer patients [56]. Kuryatov et al. characterized the function of this mutation and showed that the α5 D398 N mutation had lower Ca^2+^ permeability and greater desensitization than wild type α5 in the (α4β2)_2_α5 nAChRs [57]. Moreover, D398 N did not alter sensitivity to agonist activation of (α4β2)_2_α5 nAChRs. The (α4β2)_2_α5 AChRs promoted the release of various transmitters and were sensitive to activation and desensitization by nicotine concentrations sustained in smokers. The α5 D398 N mutation, associated with a high risk of tobacco addiction and lung cancer, exhibits decreased Ca^2+^ permeability and increased desensitization. This alteration may lead to reduced release of neurotransmitters regulated by (α4β2)_2_α5 AChRs [57].

#### 3.6.5 NMDAR mutations

Ionotropic glutamate receptors, including N-methyl-d-aspartate receptors (NMDARs), are large multi-protein complexes that form ion channels in the plasma membrane, facilitating the influx or efflux of mono- or divalent cations. These channels play critical roles in synaptic transmission, cellular migration and survival. Recently, a high prevalence of somatic mutations (W372X, Q891X, R920K, and W1271X) in GRIN2A, a subunit of NMDARs, has been identified in malignant melanoma patients [58]. The mutations disrupt the NMDAR complex between GRIN1 and GRIN2A, leading to increased anchorage-independent growth and cell migration. Mutant GRIN2A inhibits the tumor-suppressive function of the wild-type GRIN2A, promoting melanoma cell survival [58].

## 4 Conclusions and perspectives

We discuss ion channels in cancer biology and provide a summary of recent studies on ion channel mutations in different cancers. Although some mutations have been identified, only a small fraction are functionally characterized. Notably, large-scale genomic databases such as COSMIC (Catalogue of Somatic Mutations in Cancer) and NCI/GDC (National Cancer Institute/Genomic Data Commons) have collected hundreds of ion channel mutations, highlighting an important role of ion channel mutations in the field.

So far, the role of ion channel mutations on cancer progression has focused on the effects of mutations on channel biophysical functions, whether and how these mutations influence non-conductive functions in cancer biology is poorly understood. Ion channels play a crucial role in tumorigenesis by modulating intracellular signaling pathways (e.g., PI3K/Akt, MAPK, etc.) that control cancer cell proliferation, apoptosis, migration, and metabolism. How the ion channel mutations precisely modify these signaling pathways remains to be determined. Understanding these mechanistic links will provide valuable insights into cancer biology and novel therapeutic targets. Future research is required to address these gaps.

Ion channel inhibitors, such as calcium channel blockers and potassium channel modulators, have shown potential in preclinical studies [59]. For instance, the voltage-gated sodium channel inhibitor tetrodotoxin was found to reduce metastasis in animal models. Additionally, ion channel modulators might enhance the efficacy of existing cancer therapies, such as immunotherapy, by increasing cancer cell sensitivity to these treatments, highlighting their potential as complementary therapeutic strategies. Ion channel-targeted immunotherapy represents an innovative approach to enhancing anti-tumor immune responses. By modulating ion channel activity, it can improve immune cell function, reprogram the tumor microenvironment, and overcome resistance to conventional immunotherapies. Further research and clinical trials are needed to validate these strategies and translate them into effective cancer treatments.

On the other hand, despite recent advances in the ion channel-targeted therapies, several challenges remain. First, ion channels are expressed in a wide range of tissues, raising concerns about off-target effects and toxicity, which can lead to unintended physiological consequences. Developing strategies to enhance selectivity while minimizing systemic toxicity remains a critical focus in the field. Second, the limited pharmacological success of these therapies in cancer treatment is partly due to the heterogeneity of the underlying tumor microenvironment and the diverse roles that ion channels play in cancer progression. Ion channel dysregulation varies among different tumor types and even within the same tumor, complicating the development of universal therapeutic strategies. Additionally, a key obstacle remains in achieving mutation specificity, as ion channel mutations can influence cancer progression and resistance to therapy in distinct ways. Understanding the functional consequences of specific ion channel mutations and their interactions with other oncogenic pathways is crucial for optimizing treatment efficacy.

In conclusion, the development of an ion channel mutation-targeted medicine that can assess and predict responses to targeted therapy would represent a major advancement. Such an approach would enable precision medicine strategies tailored to individual patient profiles, improving therapeutic outcomes while reducing adverse effects. Future research should focus on integrating structural biology, computational modeling, and high-throughput screening techniques to design highly selective modulators that target cancer-associated ion channel mutations. Furthermore, combination therapies that exploit the interplay between ion channel mutations and other oncogenic pathways may offer a promising avenue to enhance treatment effectiveness.

## CRediT authorship contribution statement

**Xinyu Cao:** Writing – review & editing, Writing – original draft. **Liang Yan:** Writing – review & editing, Writing – original draft. **Liang Hong:** Writing – review & editing, Writing – original draft, Supervision, Funding acquisition, Conceptualization.

## Declaration of competing interest

We have nothing to declare.

## Abbreviations

EGFR: Epidermal growth factor receptor
PTEN: Phosphatase and tensin homologue
TP53: Tumor protein P53
NSCLC: non-small cell lung cancer
ROS: reactive oxygen species
HIF: hypoxia-inducible factors
*KCNJ*: potassium inwardly rectifying channel subfamily J
GIRK: G protein-coupled inwardly rectifying potassium channel
Nav: Voltage-gated sodium channel
Cav: Voltage-gated calcium channel
Hv: Voltage-gated proton channel
TRP: Transient receptor potential channel
*CLCA*: Chloride channel calcium-activated
AChR: acetylcholine receptor
nAChR: Nicotinic acetylcholine receptor
NMDAR: N-methyl-d-aspartate receptor
